# Appropriate Sequence for Afatinib and Cisplatin Combination Improves Anticancer Activity in Head and Neck Squamous Cell Carcinoma

**DOI:** 10.3389/fonc.2018.00432

**Published:** 2018-10-05

**Authors:** Eleonore Longton, Kathleen Schmit, Maude Fransolet, François Clement, Carine Michiels

**Affiliations:** Unit of Biochemistry and cellular Biology, Namur Research Institut for LIfe Sciences, University of Namur, Namur, Belgium

**Keywords:** head and neck squamous cell carcinoma, afatinib, combined therapy, treatment schedule, cisplatin

## Abstract

Despite a better understanding in head and neck tumors pathogenesis as well as improvements in radiotherapy and surgery, locally advanced head and neck squamous cell carcinoma (HNSCC) remains of poor prognosis. One promising target is the epidermal growth factor receptor (EGFR), which is overexpressed in the majority of HNSCC and is associated to tumor progression and resistance to treatment. However, in several clinical trials, the combination of EGFR inhibitors with chemotherapy and/or radiotherapy generates moderate results. In this study, we investigated the anti-tumor activity of afatinib, an irreversible pan-EGFR inhibitor, combined to cisplatin in different schedules of exposure. For that, we used two human EGFR wild-type HNSCC cell lines and we evaluated the cytotoxicity of the two drugs combined in different sequences. The efficiency of each strategy was assessed by evaluating the effects on cell cycle distribution, DNA damage, cell death and downstream pathways of ErbB family receptors. We demonstrated that cisplatin treatment followed by afatinib exposure displayed more cytotoxic effects than the opposite timing or than simultaneous association. This higher anticancer activity is probably due to afatinib-induced cell cycle arrest, which prevents the repair of cisplatin-induced DNA damage and promotes cell death by various mechanisms including apoptosis. These data suggest the importance of an appropriate timing administration between an EGFR inhibitor and a conventional chemotherapy in order to obtain the best clinical benefit for patients with a head and neck cancer.

## Introduction

Head and neck squamous cell carcinoma (HNSCC) is the sixth most common malignancy worldwide and continues to remain of poor prognosis despite advancements in treatment options (1). Currently, standard treatment of advanced HNSCC includes surgery, radiotherapy and chemotherapy, with cisplatin being the most commonly used drug in HNSCC (2). However, despite these intensive combined modality therapies, more than 50% of patients with advanced disease develop loco-regional recurrence within 2 years, and 20–30 % of those patients develop distant metastasis (3). These data underline the urgent need for development of more effective therapeutic strategies, without increasing toxicity.

Epidermal growth factor receptor (EGFR), a member of HER (ErbB) family tyrosine kinase receptors that includes EGFR (HER1/ErbB-1), HER2/neu (ErbB-2), HER3 (ErbB-3) and HER4 (ErbB-4) (4, 5), is overexpressed in up to 90% of patients with HNSCC (6). Ligand-induced homo- or hetero-dimerization of HER family members (7) activates major downstream signaling pathways, including Ras/Raf/MAPK (8), PI3K/Akt (9) and/or PLC/PKC that are linked to cell proliferation, DNA repair, survival, and differentiation (10–13). Therefore, overexpression of EGFR enhances tumorigenesis and is associated with treatment resistance and poor prognosis (14, 15). In addition, it is postulated that EGFR signaling is activated in response to DNA damage induced by radiation therapy (16) and by chemotherapeutic agents including cisplatin (17). Several EGFR targeted therapies have been developed and approved for clinical use such as cetuximab, a monoclonal antibody (18, 19), or tyrosine kinase inhibitors (TKIs), including panitumumab, erlotinib, and gefitinib. Particularly in locally advanced HNSCC, cetuximab combined to radiotherapy or chemotherapy showed an improvement in overall survival (20, 21). However, despite the clear advantages of combined EGFR targeted therapy with radiation or chemotherapy, many patients do not respond to anti-EGFR therapeutics (22–24) or develop subsequently resistance (25). One of the potential mechanisms of intrinsic or acquired resistance implicates the upregulation/activation of other HER family receptors in the presence of single receptor inhibition (26, 27). These findings led to the development of inhibitors targeting the kinase domain of all HER family members, the most promising one being afatinib.

Afatinib, a second generation of pan-ErbB inhibitor, irreversibly binds to EGFR, HER2 and HER4, and inhibits their enzymatic activity. HER3 being a kinase-inactive, it needs hetero-dimerization with other ErbB receptors and thus, it is indirectly also blocked by afatinib (28–30). In preclinical studies, afatinib demonstrated more prolonged suppression of receptor kinase activity compared to reversible first-generation EGFR-TKIs (28, 31) and it also showed activity in tumor cells resistant to reversible EGFR inhibitors (32). Moreover, in several tumor cell lines including non-small cell lung cancer (NSCLC), pancreatic cancer and colorectal cancer, the irreversible inhibition of all ErbB family receptors by afatinib resulted in an inhibition of cellular growth and induced apoptosis (33). The U.S. Food and Drug Administration (FDA) has approved afatinib for first-line treatment of patients with locally advanced or metastatic NSCLC with activating EGFR mutations (34, 35).

Preclinical studies and clinical trials in the treatment of head and neck tumors have also demonstrated the interest of afatinib used alone or combined with cytotoxic drugs or radiation therapy (36–42). These studies suggest that afatinib may enhance the antitumor activity of cytotoxic drugs. However, the sequence of administration of afatinib and cisplatin, the most commonly used chemotherapeutic agent in HNSCC has never been addressed but could improve the therapeutic response while decreasing the toxicity. Indeed, some preclinical studies have already shown the importance of a sequential administration, particularly between chemotherapies and EGFR inhibitors (43–46).

Therefore, in this study, we assessed the effects of different sequences of afatinib combined to cisplatin on cell growth, cell cycle distribution and induction of apoptosis in Cal27 and SQD9, two EGFR wild-type HNSCC cell lines.

## Materials and methods

### Cell culture

Cal27, a human tongue squamous cell carcinoma cell line, was obtained from American Type Culture Collection (ATCC, Rockville MD, USA). SQD9, a human laryngeal squamous cell carcinoma cell line, was kindly provided by Prof. Pierre Sonveaux (Université Catholique de Louvain, Brussels, Belgium). The cells were kept in a humidified atmosphere containing 5% CO_2_ at 37°C and the medium was changed 2–3 times per week. Cells were cultured in Minimum Essential Medium (MEM) with GlutaMAX™ (Thermo Fisher Scientific, Belgium) supplemented with 10% heat-inactivated fetal bovine serum.

### Incubation with afatinib and cisplatin

Cisplatin was purchased from Sigma-Aldrich (Belgium), stored according to the manufacturer's protocol and diluted in sterile PBS. Afatinib was purchased from Selleckchem (distributed by Absource Diagnostics GmbH, München, Germany), diluted in DMSO and stored according to the manufacturer's protocol. Afatinib concentrations used in the study were derived from serial dilutions in cell culture medium.

### Cytotoxic assay

Cytotoxicity was assessed by a colorimetric assay using 3-(4,5-dimethylthiazol-2-yl)-2,5-diphenyltetrazolium bromide (MTT). Cal27 and SQD9 were seeded in 24-well plates at a density of 40,000 cells per well and were incubated at 37°C with 5% CO_2_ for 48 h with various concentrations of afatinib alone (range, 0–100 nM/L) and cisplatin alone (range, 0–100 μM/L) to obtain inhibitory concentration (IC) values. MTT solution was prepared at a concentration of 2.5 mg/mL in phosphate-buffered saline and 500 μL were added per well. After 2 h, media and MTT solution were removed before adding lysis buffer. 1 h later, the optic density was read with a microplate spectrophotometer at 570 nm. The MTT test measures the number of metabolically active (viable) cells. Cell growth inhibition was expressed as the percentage of absorbance of the different conditions at 48 h compared with control culture at time 0. Regarding cytotoxicity effect of the different sequences for 48 h, we decided to use the cisplatin and afatinib concentrations at IC_20_ (i.e., concentration causing 20% of growth inhibitor) in order to keep an adequate number of cells in each well. The IC_20_ for afatinib was 10 nM/L and 15 nM/L and the IC_20_ for cisplatin was 15 μM/L and 20 μM/L for Cal27 and SQD9 respectively.

**Figure 1:**
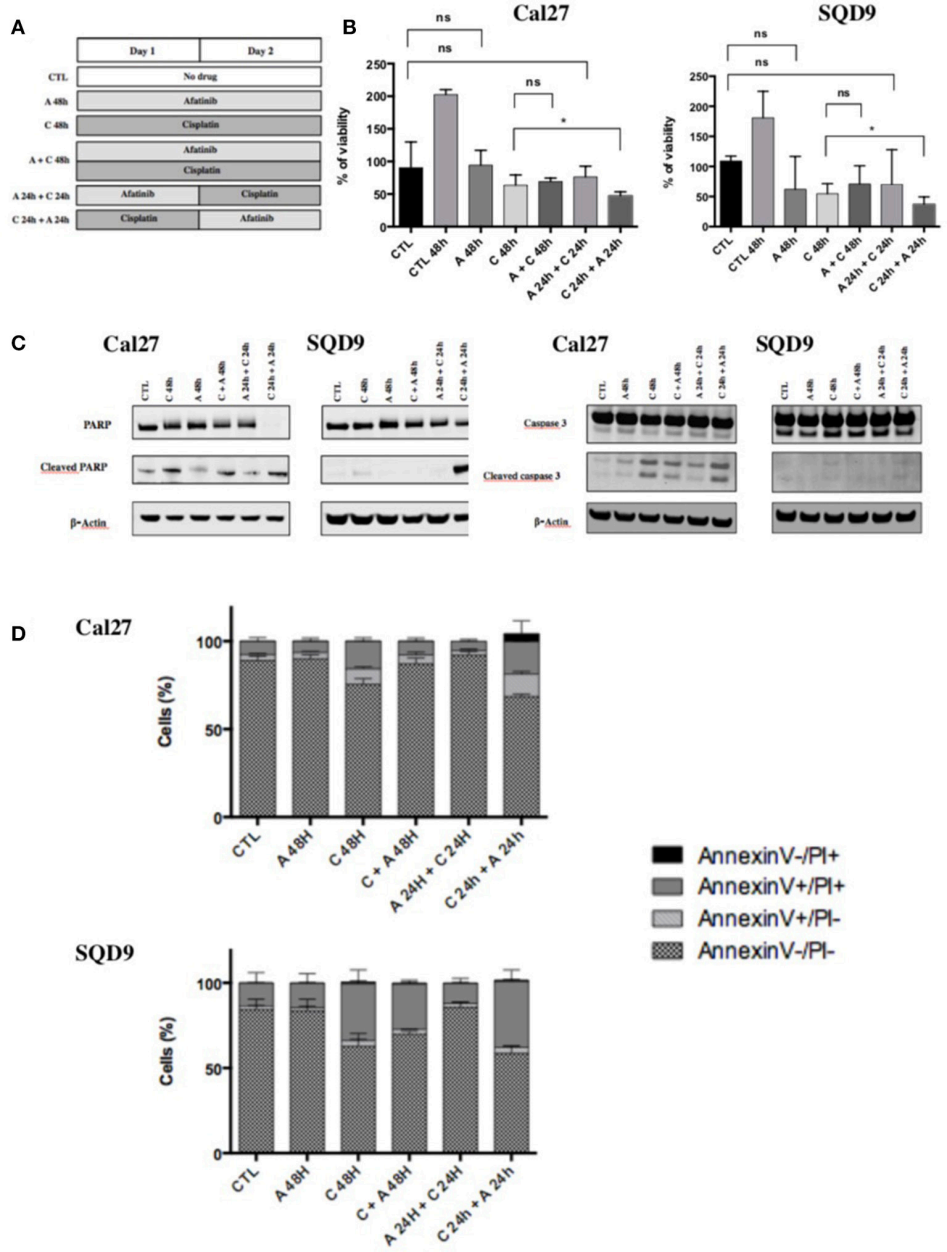
Effects of afatinib and cisplatin on the growth and apoptotic cell death. **(A)** Schematic representation of the six different conditions tested with afatinib and/or cisplatin on Cal27 and SQD9 cells. Cells were incubated for 48 h without drug (CTL), or with afatinib alone (A 48 h), with cisplatin alone (C 48h), or with the two drugs simultaneously (A + C 48 h). Cells were also incubated with drugs added sequentially: afatinib was added for 24 h before being replaced by cisplatin for 24 h (A 24 h + C 24 h), or cisplatin was added for 24 h and followed by afatinib for 24 h (C 24 h + A 24 h). **(B)** Cal27 and SQD9 cells were treated with the different combinations of afatinib and/or cisplatin for 48 h and viable cell number was analyzed by MTT assay. Data are represented as median ± interquartile range (*n* = 6) with reference to untreated control at time 0 (harvested after 24 h of culture). Mann Whitney statistical analysis were performed, *ns: p* > *0.05*, *p<*0.05*. **(C)** After 48 h of incubation with the different sequences of afatinib and/or cisplatin, Cal27, and SQD9 cell total proteins were collected. The abundance of total and cleaved forms of PARP and caspase 3 were assessed by western blot analysis with β-actin used as a loading control. **(D)** Cal27 and SQD9 cells were incubated with the different sequences of afatinib and/or cisplatin for 48 h. After the incubation, cells were harvested with trypsin/EDTA and stained using Annexin V-FITC (AnnV) and propidium iodine (PI) to detect apoptosis. The results were analyzed by flow cytometry. Cells were divided in 4 groups: Ann–/PI- for viable cells, AnnV+/PI– for cells in early apoptosis phase, AnnV+/PI+ for cells in late apoptosis, AnnV–/PI+ for cells in necrosis phase. Data are represented as mean ± SD of triplicate.

Six differents conditions of incubation between afatinib and cisplatin were tested (Figure 1A):

(1) Cells were incubated for 48 h in complete medium without drug (CTL);
(2) Cells were incubated for 48 h in complete medium with afatinib alone (A 48 h);
(3) Cells were incubated for 48 h in complete medium with cisplatin alone (C 48 h);
(4) Cells were incubated for 48 h in complete medium with the two drugs simultaneously (A + C 48 h);
(5) Cells were incubated for 48 h in complete medium with afatinib for 24 h immediately by cisplatin for 24 h (A 24 h + C 24 h);
(6) Cells were incubated for 48 h in complete medium with cisplatin for 24 h immediately by afatinib for 24 h (C 24 h + A 24 h).

In each experiment, triplicates were performed for each condition (*n* = 3) and the assay was repeated in six independent experiments to avoid any bias.

### Protein extraction and western blot analysis

Cells were seeded in 25 cm^2^ polystyrene flasks (Corning) at a density of 520 000 cells per flask. 24 h later, the medium was removed and replaced by the different solutions for 48 h. Cells were harvested, lyzed, and western blot analyses were performed as described previously by Sermeus and al. (4, 47). Primary antibodies used were reported in Table 1. Finally, the membranes were scanned with the Odyssey Infrared Imager (Li-Cor Biosciences). The fluorescence was quantified using the imagery software Odyssey V3.0 from the Odyssey Infrared Imager (Li-Cor Biosciences).

**Table 1:** Antibodies used for western blot analyses.

**Protein**	**Primary antibody**	**Secondary antibody**
EGFR	Anti-EGFR (Rabbit, Cell Signaling Technology, #4267, 1/1000)	IRDye 800CW (Anti-rabbit, Li-Cor Biosciences 926-32211, 1/10000)
Phospho-EGFR (Tyr1173)	Anti-phospho-EGFR (Rabbit, Invitrogen, #44794G, 1/1000)	IRDye 800CW (Anti-rabbit, Li-Cor Biosciences 926-32211, 1/10000)
ERK 1/2 p44/42	Anti-ERK (Rabbit, Cell Signaling Technology, #9102, 1/1000)	IRDye 800CW (Anti-rabbit, Li-Cor Biosciences 926-32211, 1/10000)
Phospho-ERK 1/2 p44/42 (Thr202/Tyr204)	Anti-phospho-ERK (Mouse, Cell Signaling Technology, #9106, 1/1000)	IRDye 680RD (Anti-mouse, Li-Cor Biosciences 926-68070, 1/10000)
AKT	Anti-AKT (Rabbit, Cell Signaling Technology, #9272, 1/1000)	IRDye 800CW (Anti-rabbit, Li-Cor Biosciences 926-32211, 1/10000)
Phospho-AKT (Ser473)	Anti-phospho-AKT (Rabbit, Cell Signaling Technology, #9271, 1/1000)	IRDye 800CW (Anti-rabbit, Li-Cor Biosciences 926-32211, 1/10000)
Caspase 3	Anti-Caspase 3 (Rabbit, Cell Signaling Technology, #9662, 1/2000)	IRDye 800CW (Anti-rabbit, Li-Cor Biosciences 926-32211, 1/10000)
Cleaved Caspase 3	Anti-Cleaved Caspase 3 (Rabbit, Cell Signaling Technology, #9664, 1/2000)	IRDye 800CW (Anti-rabbit, Li-Cor Biosciences 926-32211, 1/10000)
PARP	Anti-PARP (Mouse, BD Biosciences 51-6639GR, 1/1000)	IRDye 680RD (Anti-mouse, Li-Cor Biosciences 926-68070, 1/10000)

### Cell cycle and apoptosis assays by flow cytometry

Cells were seeded in 25 cm^2^ polystyrene flasks at a density of 520 000 cells per flask. For cell cycle analysis, two different concentrations of afatinib were tested. After 24 h of incubation, cells were collected by trypsinization, fixed with 70% cold ethanol and stored at −20°C. DNA staining was performed with a solution containing RNase (5 μg/ml) and 7-AAD (0.05 μg/μl).

Induction of apoptotic cell death by the different sequences of incubation with afatinib and/or cisplatin was investigated using Annexin V-FITC/Propidium Iodide assay (BD Biosciences).

Analysis was performed using a BD Bioscience FACSCalibur flow cytometer while data were processed and analyzed with ModFit 4.0 (Verity Software House). Three independent experiments were performed.

### Immunofluorescence labeling and confocal microscopy

Cells were seeded on glass coverslips in 24-well plates (Costar) at a density of 40,000 cells per well. 24 h later, the medium was removed and replaced for the different sequences of incubation with afatinib and/or cisplatin. After 48 h of incubation, the medium was removed and cells were fixed, permeabilized, and labeled following the procedure described previously (48). The primary antibody used was rabbit anti-phospho-histone H2AX (9664, cell signaling, Leiden, Netherlands), diluted at 1:400 in bovine serum albumin (BSA) 2% with phosphate buffer saline (PBS) and incubated overnight at 4°C in dark. The secondary antibody used was Alexia 488 nm anti-rabbit diluted at 1:1000 (Fisher Scientific). Nuclei were stained with Hoechst (Thermo Fisher Scientific H-21491) at 2 μg/mL at room temperature in the dark for 1 h. The coverslips were finally mounted on slides in Mowiol mounting solution (Sigma) warmed at 57°C. Slides were kept at 4°C to be observed later under a confocal laser scanning fluorescence microscope (SP5, Leica) with a constant photomultiplier.

### Statistics

Statistical analysis of the data was performed using Prism 6.04 (GraphPad Software, Inc., La Jolla, CA, USA). Due to the lack of a normal distribution and the number of measurements, data regarding cytotoxicity were evaluated using a nonparametric Mann-Whitney U test. All of the experiments were repeated at least three times. *P* ≤ 0.05 was considered to indicate a statistically significant difference.

## Results

### Sequence-dependent antiproliferative effects of afatinib and cisplatin in Cal27 and SQD9 cell lines

To determine whether an increase in the antiproliferative activity could be obtained by an appropriate schedule of cisplatin and afatinib combination, different treatment sequences were tested in Cal27 and SQD9 cells (Figure 1A). Cisplatin and afatinib inhibitory concentration (IC) values were determined by MTT assay in the two cell lines (Supplementary Data 1). In order to study the cytotoxic effect of the different combinations between afatinib and/or cisplatin with an adequate number of viable cells, we decided to use the cisplatin and afatinib concentrations at IC_20_ (i.e., concentration causing 20% of growth inhibition). The IC_20_ for afatinib was 10 nM/L and 15 nM/L and the IC_20_ for cisplatin was 15 μM/L and 20 μM/L for Cal27 and SQD9 cells respectively. In both cell lines, we observed that exposure to afatinib alone (A 48 h) and afatinib followed by cisplatin (A 24 h + C 24 h) induced a cytostatic effect after 48 h. A cytotoxic effect was observed with exposure to cisplatin for 48 h (C 48 h), without any significant difference when afatinib was added to cisplatin simultaneously (C + A 48 h) compared to cisplatin alone. However, when cisplatin was incubated 24 h before afatinib (C 24 h + A 24 h), we observed the most important cytotoxicity, which was statistically significant compared to cisplatin alone (Figure 1B).

In order to know if the cytotoxicity detected by the MTT assay is due to apoptosis, the abundance of cleaved PARP and cleaved caspase 3 has been investigated by western blot in Cal27 and SQD9 cells. The exposure to cisplatin alone for 48 h (C 48 h) induced a high level of cleaved PARP and cleaved caspase 3. Cleaved PARP and cleaved caspase 3 levels were lower when cisplatin and afatinib were added simultaneously (C + A 48 h) or when afatinib was added before cisplatin (A 24 h + C 24 h; Figure 1C, quantification in Supplementary Data 2). However, when afatinib was added 24 h after cisplatin (C 24 h + A 24 h), we observed a more important cleavage of PARP and a similar cleaved caspase 3 level than cisplatin alone in both cell lines. These results were confirmed using the Annexin V-FITC flow cytometric assay. Cells were divided in 4 groups: Ann-/PI- for viable cells, AnnV+/PI- for cells in early apoptosis phase, AnnV+/PI+ for cells in late apoptosis, AnnV-/PI+ for cells in necrosis phase (Supplementary Data 3). We have not observed any significant increase in apoptotic cell death when cells were incubated with afatinib alone for 48 h or with afatinib added 24 h before cisplatin, compared to untreated cells. In contrast, cisplatin exposure for 48 h induced an important increase in late apoptosis. This induction of apoptotic cell death by cisplatin was higher when afatinib exposure followed the cisplatin incubation (Figure 1D and Supplementary Data 3).

### Biological mechanisms underlying the sequence-dependent effect of afatinib and cisplatin

**Figure 2:**
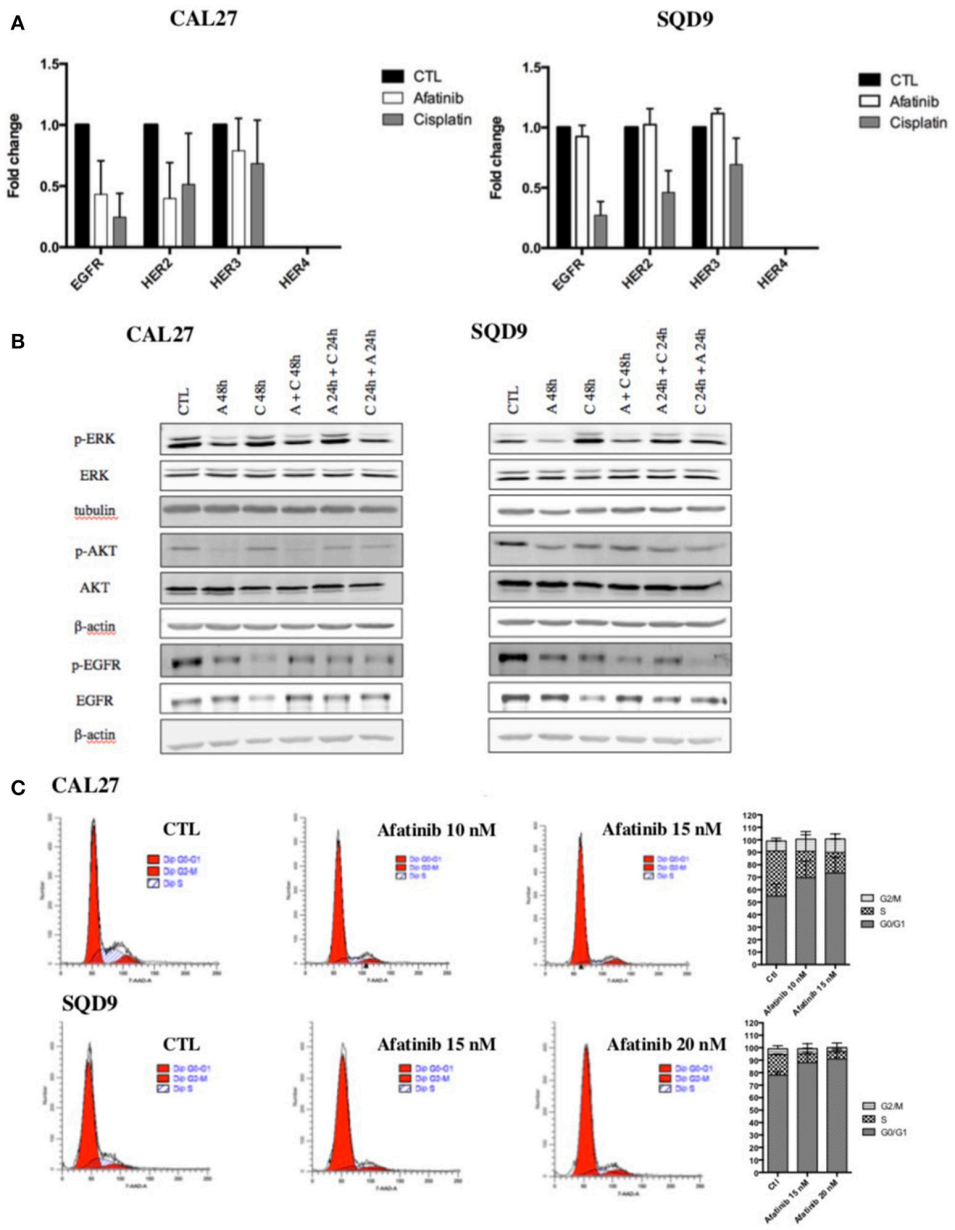
Effects of afatinib and cisplatin incubation on ErbB family receptor mRNA expression, EGFR signaling pathways and cell cycle distribution in Cal27 and SQD9 cells**. (A)** Cells were incubated with cisplatin or afatinib at IC_20_ for 24 h. Total RNA was extracted and reverse transcription was performed before mRNA expression level analysis by RT-qPCR. 23 kDa was used as housekeeping gene. Graphs showed the values of fold induction compared to cells incubated with no drug (CTL). Values are presented as mean ± SD (*n* = 3; **B**) Cells were incubated with the different sequences of cisplatin and/or afatinib for 48 h. Cell lysates were analyzed by western blot analysis. β-actin was used as a loading control. **(C)** Cal27 cells were incubated with afatinib at 10 nM and 15 nM and SQD9 cells were exposed to afatinib at 15 nM and 20 nM for 24 h. Then, cells were fixed and stained with 7-AAD and DNA content was measured by flow cytometry analysis. Cells were divided in 3 groups: G_0_/G_1_ phase (2n), S phase (2n−4n) and G_2_/M phase (4n). One representative experiment is shown for each cell line. Graphs show the percentage of cells in each cell cycle phase. Values are presented as mean ± SD (*n* = 3).

In order to explain the molecular mechanisms underlying the sequence-dependent antiproliferative effects of afatinib and cisplatin in Cal27 and SQD9 cells, we investigated the effect of each drug on EGFR signaling pathways. First, afatinib being an irreversible ErbB family blocker, its effects on the mRNA expression of EGFR as well as the mRNA expression of other ErbB family receptors (HER2, HER3 and HER4) were evaluated in Cal27 and SQD9 cells after 24 h of incubation. We also investigated the effect of cisplatin on these ErbB receptors for each human HNSCC line after 24 h of exposure. The results showed that afatinib decreased the mRNA expression of EGFR, HER2 and HER3, in Cal27 and slightly affected the mRNA expression of these ErbB receptors in SQD9 cells (Figure 2A). Interestingly, cisplatin induced a more important decrease in the EGFR, HER2, and HER3 expression of both cell lines. HER4 is not expressed in any of these two cell lines.

Then, the effects of afatinib and/or cisplatin in different sequences on the two major EGFR downstream signaling pathways, Ras/Raf/MAPK and PI3K/Akt were analyzed. Low nanomolar concentration of afatinib inhibited the activation of EGFR, AKT, and ERK1/2 in both cell lines. As already observed with mRNA expression level, the results showed a decrease in total EGFR protein level in cisplatin-treated cells. However, an increase in EGFR phosphorylation was detected. Similarly, an activation of Akt and ERK1/2 was induced by cisplatin (Figure 2B, quantification in Supplementary Data 4).

We also assessed the effect of afatinib on cell cycle. Flow cytometry analyses were performed on SQD9 and Cal27 cells 24 h after incubation with two different concentrations of afatinib. The results showed that afatinib affected the cell cycle of Cal27 and SQD9 cells: a concentration-dependent increase in the fraction of cells in the G0/G1 phase of the cell cycle from 65 to 82% at 10 nM of afatinib and to 84% at 15 nM of afatinib for Cal27, and from 77% to 82% at 15 nM of afatinib and to 86% at 20 nM of afatinib for SQD9 was observed, with a concomitant reduction of the fraction of cells in the S and G2/M phases (Figure 2C).

**Figure 3:**
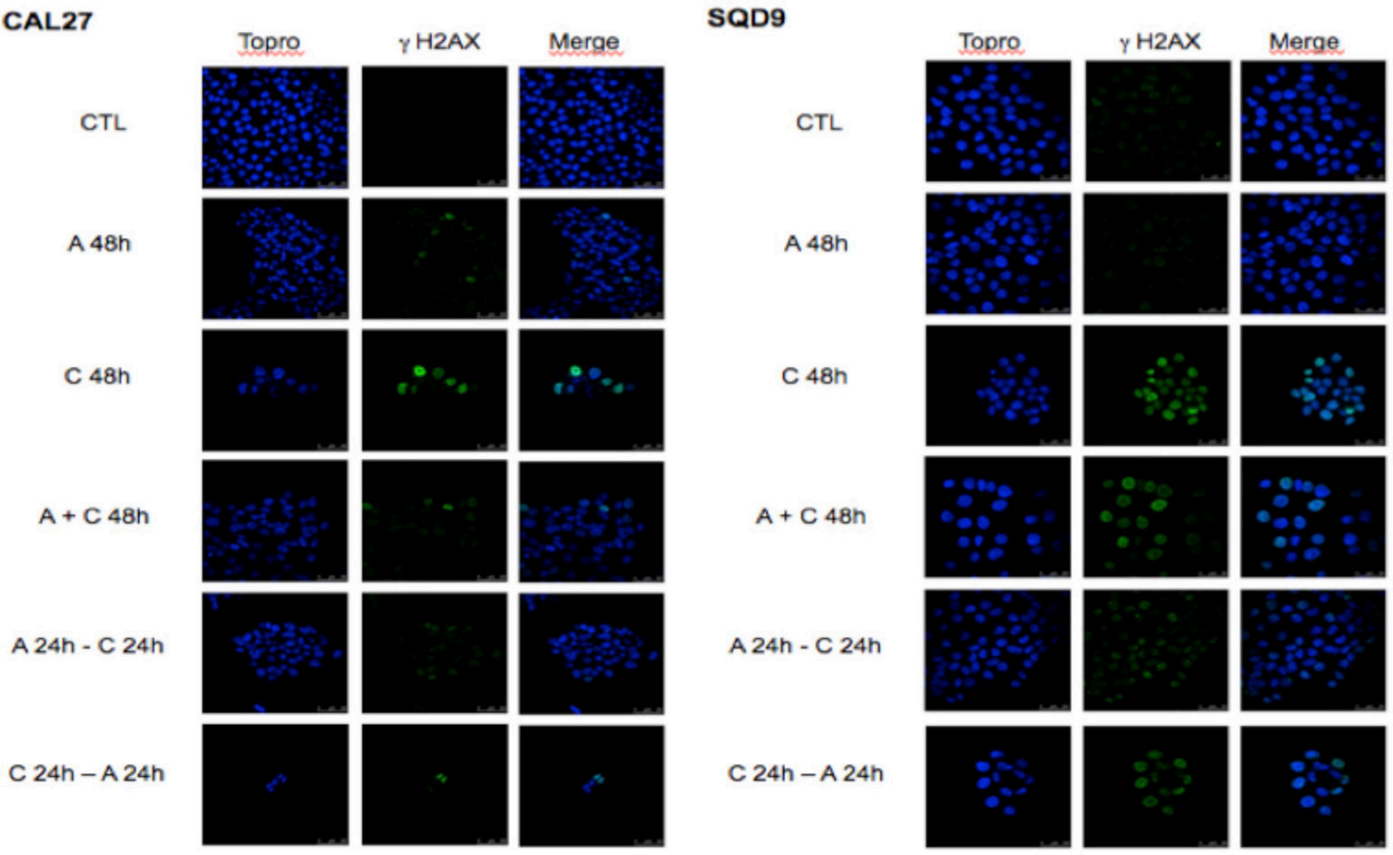
Effects of afatinib and cisplatin incubation on DNA damages. Cal27 and SQD9 cells were plated on glass cover slips and incubated and incubated with the different sequences of cisplatin and/or afatinib for 48 h. Then, cells were washed, labeled and observed under a confocal H2AX foci.

To study the combined effect of afatinib with cisplatin on DNA damage, phosphorylated γH2AX foci, a marker for DNA double-strand breaks, were analyzed. In both cell lines, after 48 h of incubation with afatinib (A 48h), no increase in γH2AX foci was observed compared to the untreated cells (CTL). This suggests that the cytostatic effect observed with afatinib is not related to DNA damage. Conversely, we observe a clear cytotoxicity effect induced by cisplatin incubation (C 48 h) with an important increase in the number of γH2AX foci per cell compared to untreated cells (CTL). This number of cisplatin-induced γH2AX foci decreased when afatinib was added before or simultaneously to cisplatin. However, the sequence with cisplatin followed by afitinib (C 24 h + A 24 h) appeared to be the most effective in terms of cytotoxicity and DNA damage (Figure 3).

## Discussion

EGFR overexpression is observed in many types of cancer, including head and neck, where it plays an important role in tumor proliferation, survival, vascularization, and metastasis. Moreover, EGFR is often activated in response to DNA damage caused by chemotherapy and/or radiation therapy (16, 17). This activation induces a survival response responsible for the resistance to treatment. In this situation, combining EGFR targeted drugs with cytotoxic drugs and/or radiation therapy that have different mechanisms of action could improve the therapeutic effectiveness. However, several clinical studies showed that combined anti-EGFR therapies with conventional chemotherapy and/or radiation generated conflicting results (29, 49). This can be explained by the resistance to EGFR targeted inhibitors, probably due to further upregulation/activation of the other members of the ErbB family, like HER2 and HER3 tyrosine kinases. These observations led to the development of inhibitors targeting all HER family members, the most promising one being afatinib. Afatinib is an irreversible EGFR/HER2/HER4 tyrosine kinase inhibitor. Afatinib used alone or combined with cytotoxic drugs or radiation therapy could be efficient in the treatment of head and neck tumors. A previous *in vitro* study using one single human hypopharyngeal cell line (FaDu) demonstrated that afatinib had a dose-dependent antiproliferative effect (40). Another *in vitro* study performed on five human EGFR wild-type HNSCC cell lines showed that afatinib in combination with cisplatin increased the growth inhibiting effect of this drug (36). Similar, an *in vivo* study highlighted that afatinib combined with gemcitabine had more significant antitumor effect than each drug used alone in a nasopharyngeal xenograft model (42). More recently, a study using both *in vitro* and *in vivo* models showed the interest of afatinib as a radiosensitizer in HNSCC cells by targeting cancer stem cells (38). Recently, it was also shown that afatinib maintained its cytotoxicity in cetuximab-resistant HNSCC cell lines (50). Afatinib also demonstrated a higher activity during clinical trials in comparison with other EGFR inhibitors. In a phase II study comparing the efficacy of cetuximab with afatinib in patients with platinum-refractory metastatic/recurrent HNSCC, the authors observed comparable response rates and suggested a possible effectiveness of afatinib in patients pre-treated with therapy targeting EGFR (41). In the phase III LUX-Head & Neck1 (LHN1) trial, afatinib significantly improved progression-free survival (PFS) vs. methotrexate treatment alone in recurrent and/or metastatic HNSCC patients progressing on/after platinum-based therapy, regardless of age (37, 39). Another hypothesis proposed to explain the resistance to EGFR inhibitors combined to cytotoxic drugs could be related to a schedule-dependent cytotoxic effect. Indeed, some studies, which combined chemotherapies and EGFR inhibitors, evidenced the importance of timing administration between the molecules. For example, it was already demonstrated that gemcitabine treatment followed by gefitinib was more cytotoxic than the opposite timing (43) or concomitant administration. The same results have been observed for combination of oxaliplatin and EGFR targeted treatments such as gefitinib and cetuximab (44–46).

Therefore, in this *in vitro* study, we investigated the effectiveness of afatinib, an irreversible EGFR/HER2/HER4 tyrosine kinase inhibitor, in combination with cisplatin, the most common chemotherapy used in HNSCC, in various sequences of treatment in two human EGFR wild-type HNSCC cell lines. The results showed different cytotoxic effects of cisplatin-associated-afatinib depending on the drug schedule. We observed a significant antiproliferative effect of afatinib combined to cisplatin only when SQD9 and Cal27 cells were incubated with cisplatin for 24 h followed by afatinib for 24 h. In contrast to the results of a previous *in vitro* study (36), we did not observe any influence of afatinib on the cytotoxic effect of cisplatin when these drugs were combined simultaneously. Contrariwise, initial exposure to afatinib seemed to protect cells from subsequent treatment with cisplatin.

The importance of using an appropriate sequence to combine afatinib with cisplatin can be explained by their respective effects on growth factor signaling pathways and cell cycle progression. Indeed, it is postulated that the response to cisplatin-induced DNA damages implicates Src kinase activation, which is responsible for the EGFR autophosphorylation (17). The EGFR activation induces a survival response that reduces the efficacy of cisplatin. EGFR activation after 24 and 48 h of cisplatin incubation was indeed observed, and we also observed a clear activation of Akt and ERK1/2 in CAL27 and SQD9 cells exposed to cisplatin. Afatinib, by inhibiting the tyrosine kinase activity of EGFR, leads to the inhibition of the PI3K/Akt and RAS/Raf/ERK1/2 pro-survival pathways in both cell lines (51). By these means, afatinib also establishes a G_0_/G_1_ cell cycle arrest, as demonstrated in previous studies in HNSCC (38, 52). We also observed a dose-dependent cell-cycle arrest in the G_0_/G_1_ phase after 24 h of afatinib exposure. Hence, this arrest of cells in G1 phase prevents the action of cisplatin, since this drug requires cells entering in S phase to exert its cytotoxic activity. This explains in part why afatinib antagonizes the cytotoxic activity of cisplatin when it is added simultaneously or before cisplatin incubation. Conversely, when afatinib is added 24 h after cisplatin, it decreases the fraction of cells in the S-phase, hence preventing the repair of DNA damage caused by cisplatin, which promotes cell death by different mechanisms including apoptosis. Indeed, our results showed a higher number of γH2AX foci in cisplatin-treated cells compared to control or afatinib-treated cells. The number of γH2AX foci was even higher when afatinib was added 24 h after cisplatin, suggesting that afatinib induced a blockade of the DNA repair. In parallel, more apoptotic cell death was demonstrated when the cells were incubated with cisplatin and then afatinib compared to cisplatin alone.

The objective of our study was to try to understand why the clinical trials mentioned hereabove did not improve the outcome of the patients. EGFR inhibition provokes cell cycle arrest that prevents DNA damage repair induced either by X-ray irradiation or cisplatin. It also decreases the cell repopulation between 2 fractions of radiotherapy. On the opposite, cell cycle arrest before or during the treatment may protect cells from the efficacy of these two treatments. Our results are in accordance with the clinical observations of Ang et al. (22) and the CONCERT studies and although they do not prove that EGFR-inhibition after chemo-radiotherapy would have worked better, they provide a possible explanation for the failure of these trials to meet their objective. Our results demonstrate that the sequence of the administration of the two drugs/therapies must be carefully studied in order to improve the therapeutic effectiveness. Nowadays, several clinical studies combine different therapies, including chemotherapy and radiation therapy, but also targeted therapies and immunotherapy. While they seem very promising, they have their own specific biomolecular effects. In our opinion, preclinical studies will remain an important step in the evaluation of optimal timing, duration, and dose between multiple therapies combined together. Our data support the hypothesis that afatinib is a good partner associated with cisplatin but, based on an understanding of the molecular mechanisms of each drug, demonstrate the importance of optimizing the right timing. Indeed, our results show that cisplatin followed by afatinib could be the optimal sequence in HNSCC treatment in order to obtain more tumor control. In addition, we also demonstrate that initial exposure to afatinib could protect cells from subsequent treatment with cisplatin and thus should be avoided. Although this *in vitro* study is based on two HNSCC cell lines and needs to be validated *in vivo*, it highlights the interest of translational researches, particularly important for oncologists and radiation oncologists when different therapies are combined.

## Author contributions

EL and CM designed and wrote the final draft of the manuscript. EL carried out the *in vitro* experiments and performed statistical analysis. KS assisted in the *in vitro* experiments and participated in its design. FC assisted in the flow cytometry analysis and MF participated in the western blot experiments. All authors read and approved the final version of the manuscript.

### Conflict of interest statement

The authors declare that the research was conducted in the absence of any commercial or financial relationships that could be construed as a potential conflict of interest.

## Supplementary material

Supplementary data 1 Cisplatin and afatinib inhibitory concentration (IC) values were determined by MTT assay in the two cell lines.

Supplementary data 2 Quantification of cleaved PARP relative to uncleaved PARP after normalization to β-actin for the different sequences of afatinib and/or cisplatin incubation in Cal27 and SQD9 cells. Values are presented as mean ± SD (*n* = 3).

Supplementary data 3 Cal27 and SQD9 cells were incubated with the different sequences of afatinib and/or cisplatin for 48 h. After the incubation, cells were labeled using Annexin V-FITC (AnnV) and propidium iodine (PI) to detect apoptosis by flow cytometry. Graphs represent the apoptotic cells in early and late phases for both cell lines.

Supplementary data 4 Quantification of phosphorylated EGFR, ERK and Akt relative to their total form after normalization to β-actin or tubulin for the different sequences of afatinib and/or cisplatin incubation in Cal27 and SQD9 cells. Values are presented as mean ± SD (*n* = 3).

## References

[1] Siegel R Naishadham D Jemal A Cancer statistics, 2012. CA Cancer J Clin. (2012) 62:10–29. 10.3322/caac.20138 22237781

[2] Gregoire V Lefebvre JL Licitra L Felip E. Squamous cell carcinoma of the head and neck: EHNS-ESMO-ESTRO Clinical Practice Guidelines for diagnosis, treatment and follow-up. Ann Oncol. (2010) 21 (Suppl. 5):v184–6. 10.1093/annonc/mdq185 20555077

[3] Vermorken JB Specenier P. Optimal treatment for recurrent/metastatic head and neck cancer. Ann Oncol. (2010) 21 (Suppl. 7):vii252-61. 10.1093/annonc/mdq453 20943624

[4] Sermeus A Cosse JP Crespin M Mainfroid V de Longueville F Ninane N. Hypoxia induces protection against etoposide-induced apoptosis: molecular profiling of changes in gene expression and transcription factor activity. Mol Cancer (2008) 7:27. 10.1186/1476-4598-7-27 18366759 PMC2330149

[5] Yarden Y Sliwkowski MX. Untangling the ErbB signalling network. Nat Rev Mol Cell Biol. (2001) 2:127–37. 10.1038/35052073 11252954

[6] Grandis JR Tweardy DJ. Elevated levels of transforming growth factor alpha and epidermal growth factor receptor messenger RNA are early markers of carcinogenesis in head and neck cancer. Cancer Res. (1993) 53:3579–84. 8339264

[7] Schlessinger J. Cell signaling by receptor tyrosine kinases. Cell (2000) 103:211–25. 10.1016/S0092-8674(00)00114-8 11057895

[8] Lewis TS Shapiro PS Ahn NG. Signal transduction through MAP kinase cascades. Adv Cancer Res. (1998) 74:49–139. 10.1016/S0065-230X(08)60765-4 9561267

[9] Vivanco I Sawyers CL. The phosphatidylinositol 3-Kinase AKT pathway in human cancer. Nat Rev Cancer (2002) 2:489–501. 10.1038/nrc839 12094235

[10] Albanell J Codony-Servat J Rojo F Del Campo JM Sauleda S Anido J. Activated extracellular signal-regulated kinases: association with epidermal growth factor receptor/transforming growth factor alpha expression in head and neck squamous carcinoma and inhibition by anti-epidermal growth factor receptor treatments. Cancer Res. (2001) 61:6500–10. 11522647

[11] Testa JR Bellacosa A. AKT plays a central role in tumorigenesis. Proc Natl Acad Sci USA. (2001) 98:10983–5. 10.1073/pnas.211430998 11572954 PMC58668

[12] Chen DJ Nirodi CS. The epidermal growth factor receptor: a role in repair of radiation-induced DNA damage. Clin Cancer Res. (2007) 13(22 Pt 1):6555–60. 10.1158/1078-0432.CCR-07-1610 18006754

[13] Huang SM Harari PM. Modulation of radiation response after epidermal growth factor receptor blockade in squamous cell carcinomas: inhibition of damage repair, cell cycle kinetics, and tumor angiogenesis. Clin Cancer Res. (2000) 6:2166–74. 10873065

[14] Nijkamp MM Span PN Bussink J Kaanders JH. Interaction of EGFR with the tumour microenvironment: implications for radiation treatment. Radiother Oncol. (2013) 108:17–23. 10.1016/j.radonc.2013.05.006 23746695

[15] Agulnik M New approaches to EGFR inhibition for locally advanced or metastatic squamous cell carcinoma of the head and neck (SCCHN). Med Oncol. (2012) 29:2481–91. 10.1007/s12032-012-0159-2 22252310 PMC3466428

[16] Peter RU Beetz A Ried C Michel G van Beuningen D Ruzicka T. Increased expression of the epidermal growth factor receptor in human epidermal keratinocytes after exposure to ionizing radiation. Radiat Res. (1993) 136:65–70. 10.2307/3578641 8210340

[17] Benhar M Engelberg D Levitzki A. Cisplatin-induced activation of the EGF receptor. Oncogene (2002) 21:8723–31. 10.1038/sj.onc.1205980 12483525

[18] Specenier P Vermorken JB. Biologic therapy in head and neck cancer: a road with hurdles. ISRN Oncol. (2012) 2012:163752. 10.5402/2012/163752 22745915 PMC3382358

[19] Huang SM Bock JM Harari PM. Epidermal growth factor receptor blockade with C225 modulates proliferation, apoptosis, and radiosensitivity in squamous cell carcinomas of the head and neck. Cancer Res. (1999) 59:1935–40. 10213503

[20] Vermorken JB Mesia R Rivera F Remenar E Kawecki A Rottey S. Platinum-based chemotherapy plus cetuximab in head and neck cancer. N Engl J Med. (2008) 359:1116–27. 10.1056/NEJMoa0802656 18784101

[21] Bonner JA Harari PM Giralt J Azarnia N Shin DM Cohen RB. Radiotherapy plus cetuximab for squamous-cell carcinoma of the head and neck. N Engl J Med. (2006) 354:567–78. 10.1056/NEJMoa053422 16467544

[22] Ang KK Zhang Q Rosenthal DI Nguyen-Tan PF Sherman EJ Weber RS. Randomized phase III trial of concurrent accelerated radiation plus cisplatin with or without cetuximab for stage III to IV head and neck carcinoma: RTOG 0522. J Clin Oncol. (2014) 32:2940–50. 10.1200/JCO.2013.53.5633 25154822 PMC4162493

[23] Giralt J Trigo J Nuyts S Ozsahin M Skladowski K Hatoum G. Panitumumab plus radiotherapy versus chemoradiotherapy in patients with unresected, locally advanced squamous-cell carcinoma of the head and neck (CONCERT-2): a randomised, controlled, open-label phase 2 trial. Lancet Oncol. (2015) 16:221–32. 10.1016/S1470-2045(14)71200-8 25596659

[24] Mesia R Henke M Fortin A Minn H Yunes Ancona AC Cmelak A. Chemoradiotherapy with or without panitumumab in patients with unresected, locally advanced squamous-cell carcinoma of the head and neck (CONCERT-1): a randomised, controlled, open-label phase 2 trial. Lancet Oncol. (2015) 16:208–20. 10.1016/S1470-2045(14)71198-2 25596660

[25] Arteaga CL. EGF receptor as a therapeutic target: patient selection and mechanisms of resistance to receptor-targeted drugs. J Clin Oncol. (2003) 21(Suppl. 23):289s−91s. 10.1200/JCO.2003.10.523 14645415

[26] Wheeler DL Huang S Kruser TJ Nechrebecki MM Armstrong EA Benavente S. Mechanisms of acquired resistance to cetuximab: role of HER (ErbB) family members. Oncogene (2008) 27:3944–56. 10.1038/onc.2008.19 18297114 PMC2903615

[27] Vlacich G Coffey RJ. Resistance to EGFR-targeted therapy: a family affair. Cancer Cell (2011) 20:423–5. 10.1016/j.ccr.2011.10.006 22014569 PMC3616495

[28] Li D Ambrogio L Shimamura T Kubo S Takahashi M Chirieac LR. BIBW2992, an irreversible EGFR/HER2 inhibitor highly effective in preclinical lung cancer models. Oncogene (2008) 27:4702–11. 10.1038/onc.2008.109 18408761 PMC2748240

[29] Yap TA Vidal L Adam J Stephens P Spicer J Shaw H. Phase I trial of the irreversible EGFR and HER2 kinase inhibitor BIBW 2992 in patients with advanced solid tumors. J Clin Oncol. (2010) 28:3965–72. 10.1200/JCO.2009.26.7278 20679611

[30] Solca F Dahl G Zoephel A Bader G Sanderson M Klein C. Target binding properties and cellular activity of afatinib (BIBW 2992), an irreversible ErbB family blocker. J Pharmacol Exp Ther. (2012) 343:342–50. 10.1124/jpet.112.197756 22888144

[31] Katakami N Atagi S Goto K Hida T Horai T Inoue A. LUX-Lung 4: a phase II trial of afatinib in patients with advanced non-small-cell lung cancer who progressed during prior treatment with erlotinib, gefitinib, or both. J Clin Oncol. (2013) 31:3335–41. 10.1200/JCO.2012.45.0981 23816963

[32] Kwak EL Sordella R Bell DW Godin-Heymann N Okimoto RA Brannigan BW. Irreversible inhibitors of the EGF receptor may circumvent acquired resistance to gefitinib. Proc Natl Acad Sci USA. (2005) 102:7665–70. 10.1073/pnas.0502860102 15897464 PMC1129023

[33] Modjtahedi H Cho BC Michel MC Solca F. A comprehensive review of the preclinical efficacy profile of the ErbB family blocker afatinib in cancer. Naunyn Schmiedebergs Arch Pharmacol. (2014) 387:505–21. 10.1007/s00210-014-0967-3 24643470 PMC4019832

[34] Dungo RT Keating GM. Afatinib: first global approval. Drugs (2013) 73:1503–15. 10.1007/s40265-013-0111-6 23982599

[35] Stevenson JP. Afatinib approval for NSCLC brings EGFR TKI story full circle. J Comm Supp Oncol. (2014) 12:2–3. 10.12788/jcso.0002 24971395

[36] Brands RC Muller-Richter UD De Donno F Seher A Mutzbauer G Linz C. Co-treatment of wild-type EGFR head and neck cancer cell lines with afatinib and cisplatin. Mol Med Rep. (2016) 13:2338–44. 10.3892/mmr.2016.4786 26782932

[37] Clement PM Gauler T Machiels JP Haddad RI Fayette J Licitra LF. Afatinib versus methotrexate in older patients with second-line recurrent and/or metastatic head and neck squamous cell carcinoma: subgroup analysis of the LUX-Head & Neck 1 trial. Ann Oncol. (2016) 27:1585–93. 10.1093/annonc/mdw151 27084954 PMC4959921

[38] Macha MA Rachagani S Qazi AK Jahan R Gupta S Patel A. Afatinib radiosensitizes head and neck squamous cell carcinoma cells by targeting cancer stem cells. Oncotarget (2017) 8:20961–73. 10.18632/oncotarget.15468 28423495 PMC5400558

[39] Machiels JP Haddad RI Fayette J Licitra LF Tahara M Vermorken JB Afatinib versus methotrexate as second-line treatment in patients with recurrent or metastatic squamous-cell carcinoma of the head and neck progressing on or after platinum-based therapy (LUX-Head & Neck 1): an open-label, randomised phase 3 trial. Lancet Oncol. (2015) 16:583–94. 10.1016/S1470-2045(15)70124-5 25892145

[40] Schutze C Dorfler A Eicheler W Zips D Hering S Solca F. Combination of EGFR/HER2 tyrosine kinase inhibition by BIBW 2992 and BIBW 2669 with irradiation in FaDu human squamous cell carcinoma. Strahlenther Onkol. (2007) 183:256–64. 10.1007/s00066-007-1696-z 17497097

[41] Seiwert TY Fayette J Cupissol D Del Campo JM Clement PM Hitt R A randomized, phase II study of afatinib versus cetuximab in metastatic or recurrent squamous cell carcinoma of the head and neck. Ann Oncol. (2014) 25:1813–20. 10.1093/annonc/mdu216 24928832 PMC4143093

[42] Xue C Tian Y Zhang J Zhao Y Zhan J Fang W. In vitro and in vivo efficacy of afatinib as a single agent or in combination with gemcitabine for the treatment of nasopharyngeal carcinoma. Drug Des Devel Ther. (2016) 10:1299–306. 10.2147/DDDT.S94432 27099475 PMC4821387

[43] Chun PY Feng FY Scheurer AM Davis MA Lawrence TS Nyati MK. Synergistic effects of gemcitabine and gefitinib in the treatment of head and neck carcinoma. Cancer Res. (2006) 66:981–8. 10.1158/0008-5472.CAN-05-2665 16424033

[44] Azzariti A Xu JM Porcelli L Paradiso A. The schedule-dependent enhanced cytotoxic activity of 7-ethyl-10-hydroxy-camptothecin (SN-38) in combination with Gefitinib (Iressa, ZD1839). Biochem Pharmacol. (2004) 68:135–44. 10.1016/j.bcp.2004.03.014 15183125

[45] Morelli MP Cascone T Troiani T De Vita F Orditura M Laus G. Sequence-dependent antiproliferative effects of cytotoxic drugs and epidermal growth factor receptor inhibitors. Ann Oncol. (2005) 16 (Suppl. 4):iv61–8. 10.1093/annonc/mdi910 15923432

[46] Xu JM Azzariti A Severino M Lu B Colucci G Paradiso A. Characterization of sequence-dependent synergy between ZD1839 (“Iressa”) and oxaliplatin. Biochem Pharmacol. (2003) 66:551–63. 10.1016/S0006-2952(03)00291-0 12906920

[47] Sermeus A Rebucci M Fransolet M Flamant L Desmet D Delaive E. Differential effect of hypoxia on etoposide-induced DNA damage response and p53 regulation in different cell types. J Cell Physiol. (2013) 228:2365–76. 10.1002/jcp.24409 23702906

[48] Piret JP Cosse JP Ninane N Raes M Michiels C. Hypoxia protects HepG2 cells against etoposide-induced apoptosis via a HIF-1-independent pathway. Exp Cell Res. (2006) 312:2908–20. 10.1016/j.yexcr.2006.05.018 16844113

[49] Lefebvre JL Pointreau Y Rolland F Alfonsi M Baudoux A Sire C. Induction chemotherapy followed by either chemoradiotherapy or bioradiotherapy for larynx preservation: the TREMPLIN randomized phase II study. J Clin Oncol. (2013) 31:853–9. 10.1200/JCO.2012.42.3988 23341517

[50] De Pauw I Lardon F Van den Bossche J Baysal H Fransen E Deschoolmeester V Simultaneous targeting of EGFR, HER2, and HER4 by afatinib overcomes intrinsic and acquired cetuximab resistance in head and neck squamous cell carcinoma cell lines. Mol Oncol. (2018) 12:830–54. 10.1002/1878-0261.12197 29603584 PMC5983215

[51] Kumar S Agrawal R. Next generation tyrosine kinase inhibitor (TKI): afatinib. Recent Pat Anticancer Drug Discov. (2014) 9:382–93. 10.2174/1574892809666140520114928 24844234

[52] Liu X Lv Z Zou J Liu X Ma J Wang J. Afatinib down-regulates MCL-1 expression through the PERK-eIF2alpha-ATF4 axis and leads to apoptosis in head and neck squamous cell carcinoma. Am J Cancer Res. (2016) 6:1708–19. 27648360 PMC5004074

