# Supplementary material for: Appropriate Sequence for Afatinib and Cisplatin Combination Improves Anticancer Activity in Head and Neck Squamous Cell Carcinoma
Source: Front Oncol. 2018 Oct 5;8:432. doi: 10.3389/fonc.2018.00432 (PMC6182255; doi:10.3389/fonc.2018.00432)

Supplementary data 1

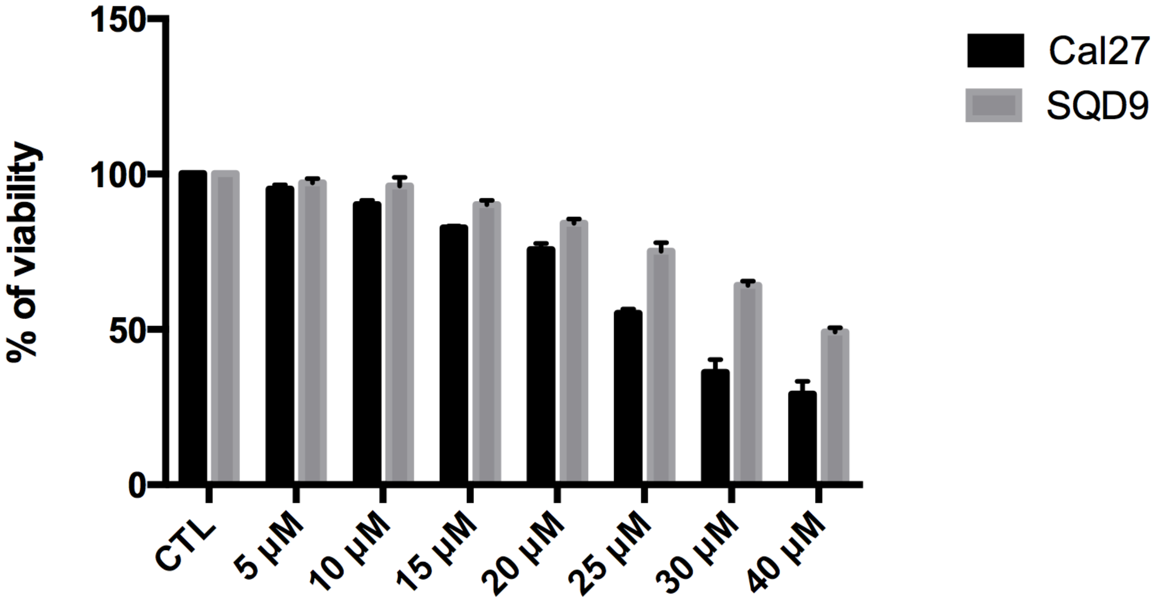

Cisplatin

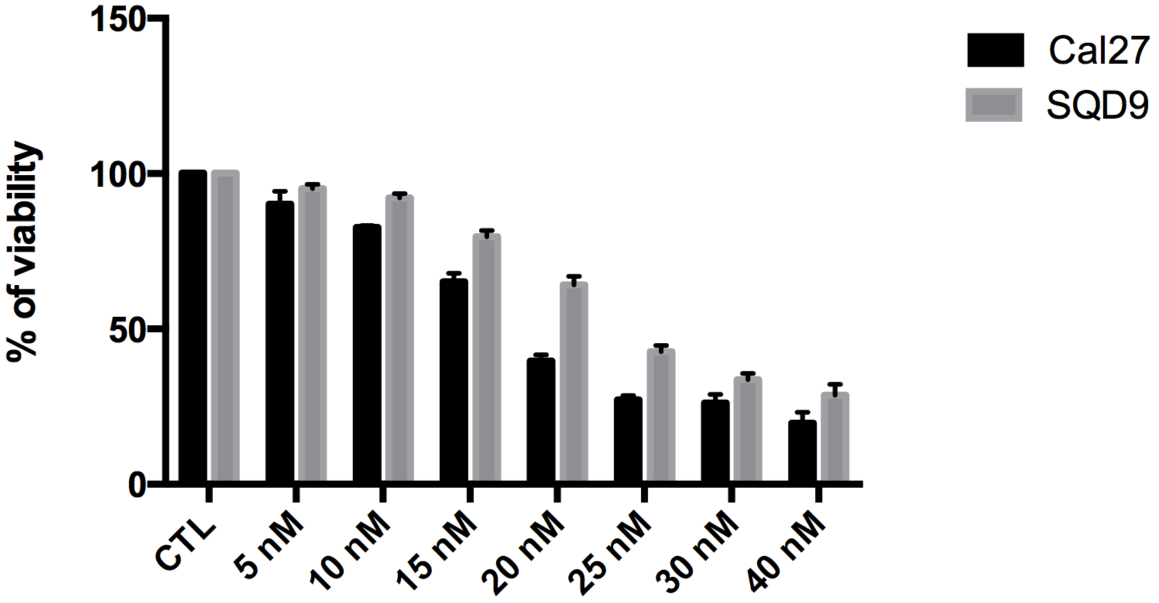

Afatinib

Supplementary data 2

Cal27

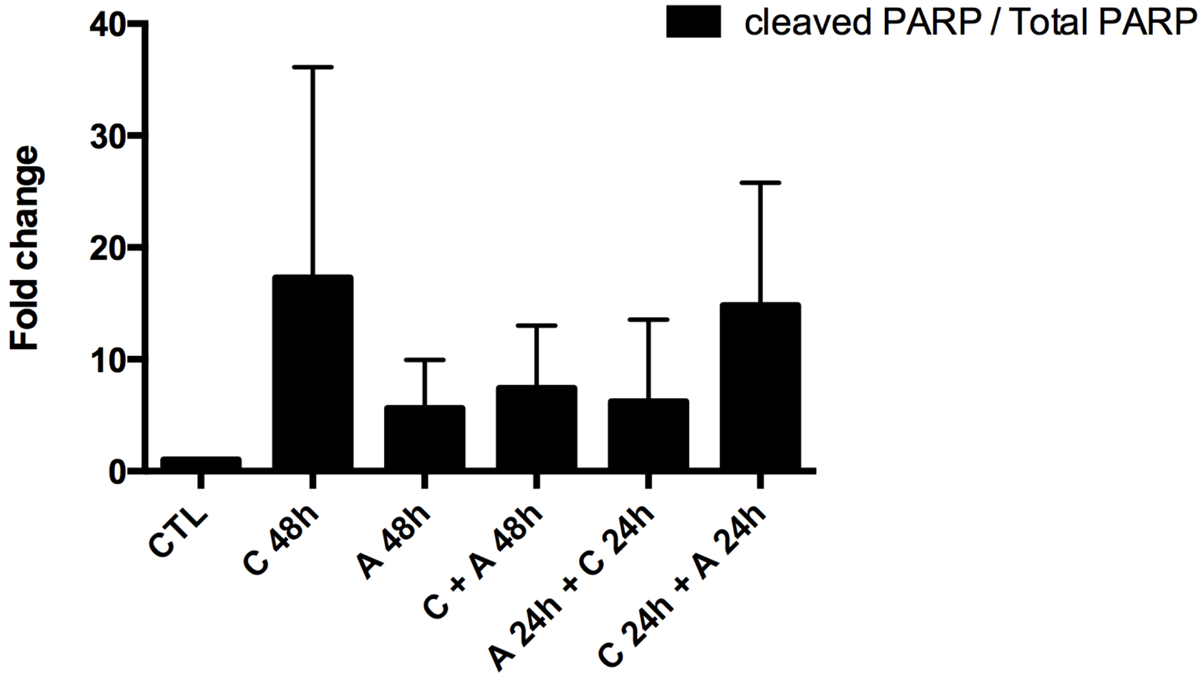

SQD9

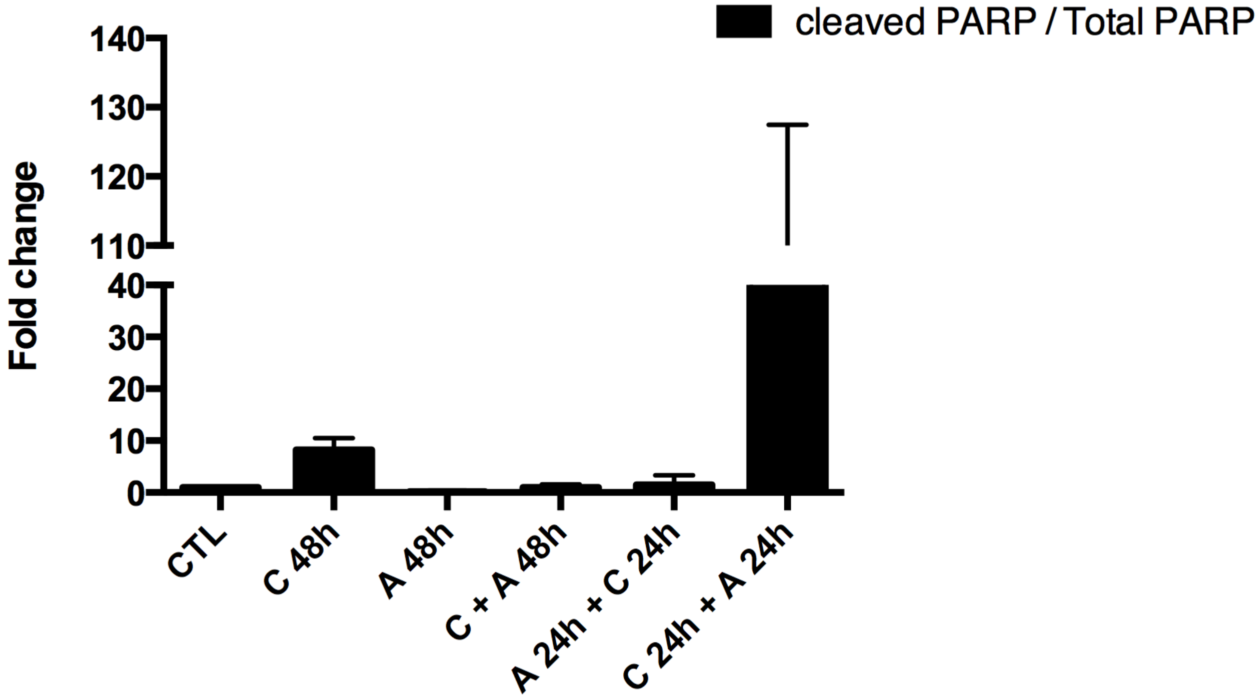

Supplementary data 3

Cal27

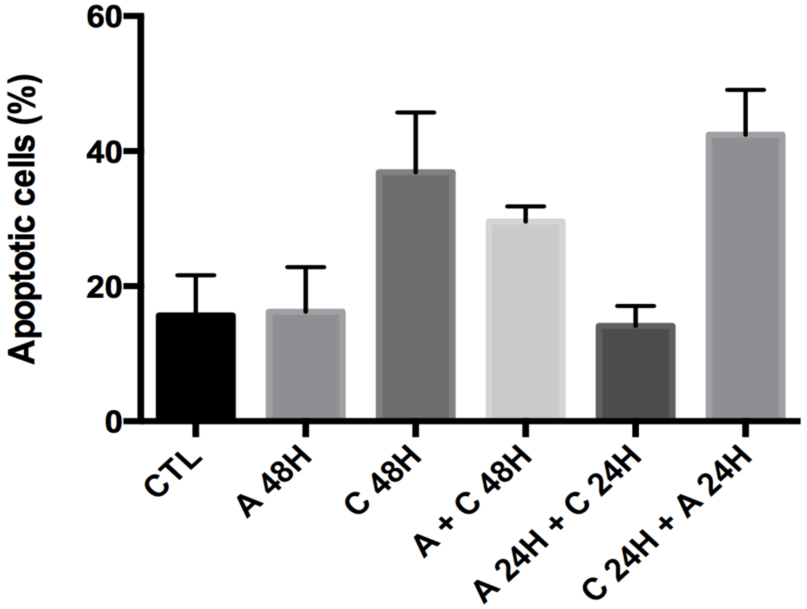

SQD9

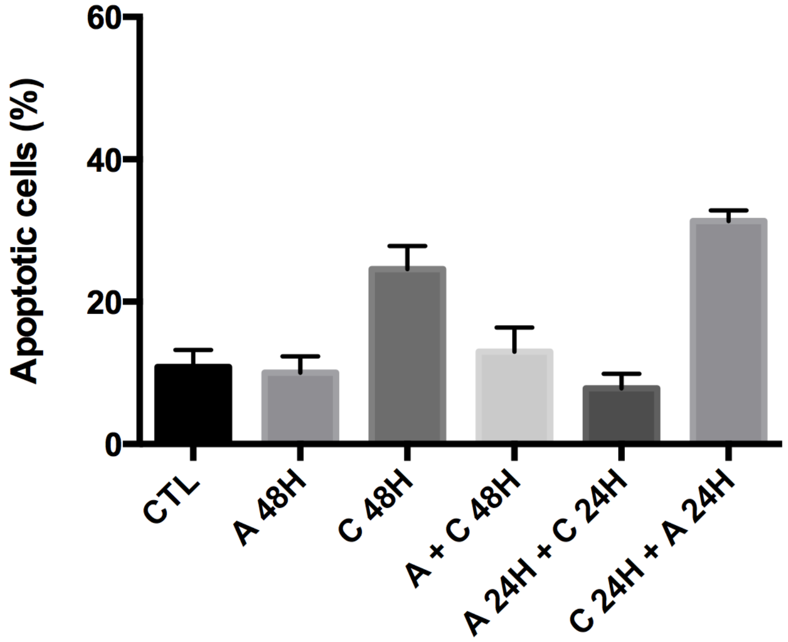

Supplementary data 4

Cal27 **SQD9**

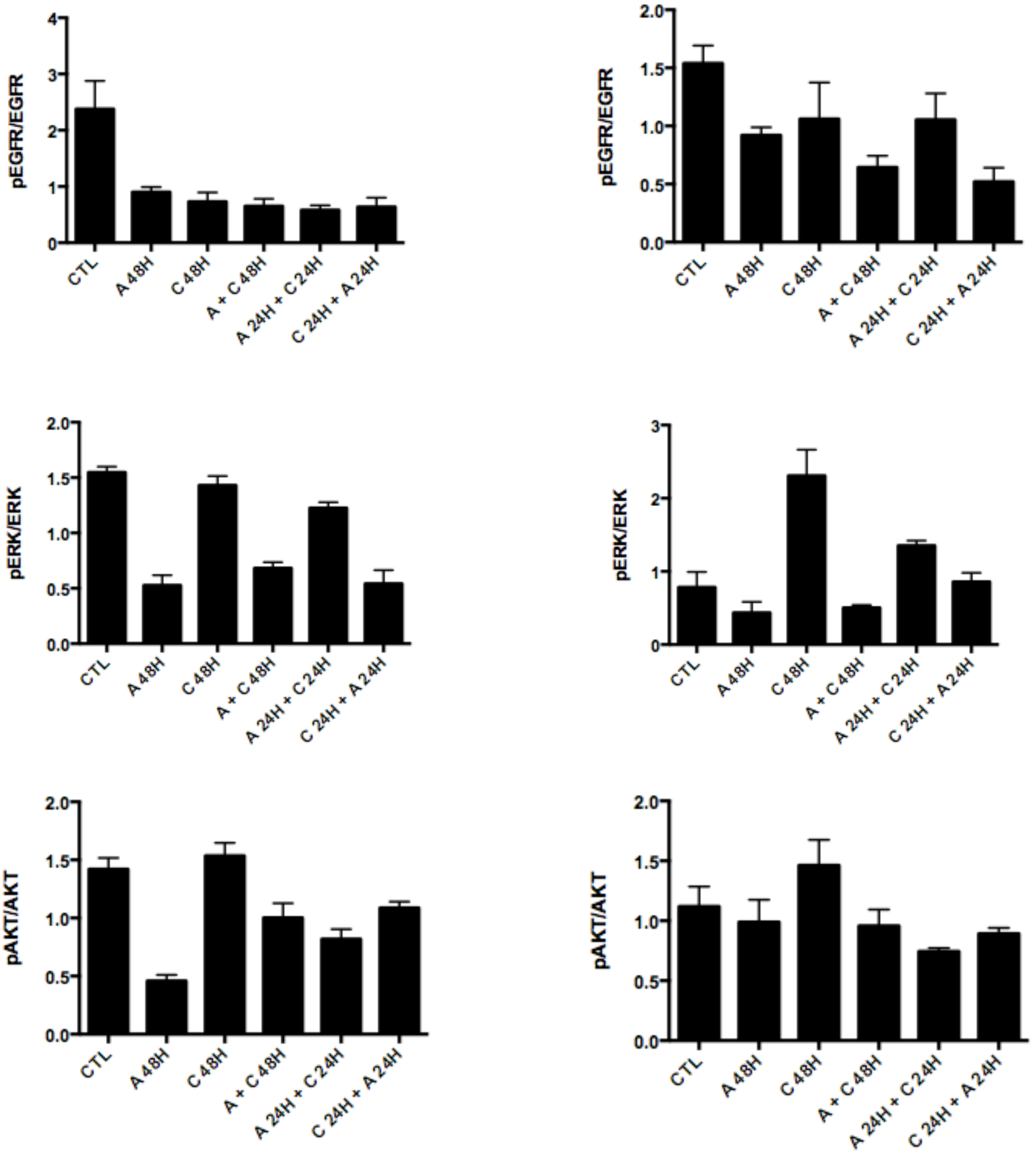

Supplement: Supplementary data 1 — Cisplatin and afatinib inhibitory concentration (IC) values were determined by MTT assay in the two cell lines. [file Data_Sheet_1.PDF]
